# The multifaceted role of post-translational modifications of LSD1 in cellular processes and disease pathogenesis

**DOI:** 10.1016/j.gendis.2024.101307

**Published:** 2024-04-15

**Authors:** Yinrui Li, Bo Wang, Yichao Zheng, Huiqin Kang, Ang He, Lijuan Zhao, Ningjie Guo, Hongmin Liu, Adil Mardinoglu, M.A.A. Mamun, Ya Gao, Xiaobing Chen

**Affiliations:** a Department of Oncology, The Affiliated Cancer Hospital of Zhengzhou University & Henan Cancer Hospital, Henan Engineering Research Center of Precision Therapy of Gastrointestinal Cancer & Zhengzhou Key Laboratory for Precision Therapy of Gastrointestinal Cancer, Zhengzhou, Henan 450008, China; b State Key Laboratory of Esophageal Cancer Prevention & Treatment, Key Laboratory of Advanced Drug Preparation Technologies, Ministry of Education of China, Key Laboratory of Henan Province for Drug Quality Control and Evaluation, Zhengzhou, Henan 450001, China; c Institute of Drug Discovery and Development, School of Pharmaceutical Sciences, Zhengzhou University, Zhengzhou, Henan 450001, China; d Henan Institute of Medical and Pharmaceutical Sciences, State Key Laboratory for Esophageal Cancer Prevention and Treatment, Zhengzhou University, Zhengzhou, Henan 450001, China; e Science for Life Laboratory, KTH – Royal Institute of Technology, Stockholm SE-100 44, Sweden; f Faculty of Dentistry, Oral & Craniofacial Sciences, Centre for Host-Microbiome Interactions, King's College London, London WC2R 2LS, UK

**Keywords:** Enzyme activity, Histone demethylase, Human diseases, LSD1, Post-translational modifications

## Abstract

Post-translational modifications (PTMs) of proteins play a crucial role in living organisms, altering the properties and functions of proteins. There are over 450 known PTMs involved in various life activities. LSD1 (lysine-specific demethylase 1) is the first identified histone demethylase that can remove monomethylation or dimethylation modifications from histone H3 lysine K4 (H3K4) and histone H3 lysine K9 (H3K9). This ability of LSD1 allows it to inhibit or activate transcription. LSD1 has been found to abnormally express at the protein level in various tumors, making it relevant to multiple diseases. As a PTM enzyme, LSD1 itself undergoes various PTMs, including phosphorylation, acetylation, ubiquitination, methylation, SUMOylation, and S-nitrosylation, influencing its activity and function. Dysregulation of these PTMs has been implicated in a wide range of diseases, including cancer, metabolic disorders, neurological disorders, cardiovascular diseases, and bone diseases. Understanding the species of PTMs and functions regulated by various PTMs of LSD1 provides insights into its involvement in diverse physiological and pathological processes. In this review, we discuss the structural characteristics of LSD1 and amino acid residues that affect its enzyme activity. We also summarize the potential PTMs that occur on LSD1 and their involvement in cellular processes. Furthermore, we describe human diseases associated with abnormal expression of LSD1. This comprehensive analysis sheds light on the intricate interplay between PTMs and the functions of LSD1, highlighting their significance in health and diseases.

## Introduction

LSD1 (lysine-specific demethylase 1), also known as KDM1A, AOF2, and BHC110, plays a key role in the regulation of gene expression because of the activity of histone demethylation. It was initially found as a nucleoprotein that binds to flavin adenine dinucleotide (FAD), and shares sequence homology with FAD-dependent amine oxidases. Subsequently, it was identified as the histone demethylase in 2004, capable of removing mono/di-methylation modifications from the fourth lysine on the N-terminus of histone H3.^1^, ^2^, ^3^ Histone modifications play a crucial role in regulating gene expression and chromatin structure. The primary function of LSD1 is removing methyl groups from lysine residues of methylated H3K4 and H3K9. This activity allows LSD1 to regulate gene expression by altering the chromatin structure. The complete demethylation process is as follows: the FAD binds to LSD1 to mediate the two-electron oxidation of lysine at the fourth or ninth position of methylated histone, the α-carbon bond oxidative cleavage of the substrate, and two electrons are transferred from the methyl carbon of methylated lysine to FAD in the form of hydride anions and generate imine intermediates, while FAD is reduced to generate FADH_2_ (fully reduced flavine adenine dinucleotide); imine intermediates are hydrolyzed via non-enzymatic processes to generate aldehydes and amines, and FADH_2_ is converted to FAD by regarding molecular oxygen as the electron acceptor with the generation of hydrogen peroxide, thus completing the cycle.^1^^,^^3^, ^4^, ^5^ Another study revealed that LSD1 demethylation does not exhibit a strong preference for monomethylation or dimethylation of H3K4. This phenomenon can be attributed to the inherent chemistry of flavin-containing amine oxidases. Substrates of LSD1 require a protonated nitrogen, which means that LSD1 cannot effectively utilize trimethylated histones as substrates.^1^^,^^6^

Post-translational modifications refer to the effect of protein levels or function by adding or removing functional groups or amino acids. In general, LSD1 could interact with several co-repressor complexes, such as CoREST (REST corepressor) and HDACs, to modulate chromatin structure and gene expression via demethylation activity. Similarly, LSD1 itself also can be subjected to post-translational modifications as a production of gene expression, such as phosphorylation, acetylation, ubiquitination, methylation, SUMOylation, and S-nitrosylation, which influence its enzymatic activity, stability, and subcellular localization.

Recently, many studies have shown that dysregulation of LSD1 protein has been implicated in various diseases, including cancer, metabolic disorders, neurological disorders, cardiovascular diseases, and bone diseases. The aberrant activity of LSD1 can lead to dysregulated gene expression and contribute to disease pathogenesis. Therefore, understanding the post-translational modifications and functions of LSD1 holds great significance in unraveling its involvement in diverse physiological and pathological processes.

In this article, we provide an overview of the structure, post-translational modifications, and related diseases of LSD1. By shedding light on the intricate interplay between LSD1 and PTMs, we aim to highlight the significance of these modifications of LSD1 in health and diseases, potentially opening new avenues for therapeutic interventions targeting LSD1 and its associated diseases.

## Structure of LSD1

**Figure 1:**
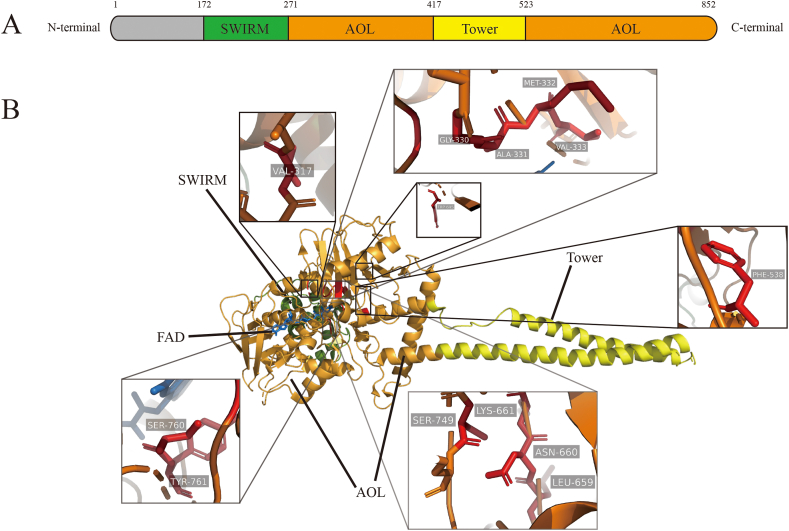
The structure of LSD1.

The LSD1 gene encodes 852 amino acids and consists of the SWIRM (Swi3/Rcs8/Moira) domain, AOL (amine oxidase-like) domain, Tower domain, and N-terminal disordered sequence, and the three key domains are highly conserved in different species (Fig. 1A, B).^7^ There is a disordered sequence consisting of 150 amino acids at the N-terminus of LSD1 containing nuclear localization signals, which mainly affects the interaction between proteins and the fragment occurs post-translational modifications, such as phosphorylation and methylation.^8^^,^^9^ In the activity assay, it was found that the activity of LSD1 without this sequence was not significantly different from that of the full length, indicating that this sequence did not affect the demethylase activity of LSD1.^4^^,^^6^^,^^10^^,^^11^ The SWIRM domain exists in many proteins that are involved in chromatin remodeling and histone modification. It is mainly composed of α-helices, mediates the interaction between proteins, and is commonly found in chromatin-related proteins.^7^^,^^11^, ^12^, ^13^, ^14^, ^15^ The SWIRM domain of LSD1 is different from other amine oxidases in its family. The N-terminus of the SWIRM domain is tightly arranged, consisting of 100 amino acids (residues 172–271), connected to a part of the amine oxidase domain (residues 271–417). The SWIRM domain of LSD1 is a six-helix bundle structure composed of α-helices, with a long helix in the center surrounded by five helixes (α1/2/3/5/6). A feature of the SWIRM domain of LSD1 is that at its C-terminus, two stranded β-sheets are additionally formed. The fold sheets protrude to the hydrophobic pocket of the AOL domain, anchoring the interaction of SWIRM and AOL.^11^ SWIRM and AOL domains are linked by an extensive network of van der Waals contracts to form a surface region, and the interface has a high degree of hydrophobicity. The interaction of the two domains results in the formation of a highly conserved cleft near the cavity of the active site,^10^ which can combine with the N-terminal tail of the H3 histone substrate.^13^ Another feature of the SWIRM domain of LSD1 is that the amino acid residues binding to DNA in the SWIRM are not conserved, and the amino acid residues of the cleft formed by the SWIRM and AOL domains are essentially invariant across mammals. It is speculated that this conserved cleft may be related to the histone tail substrate.^10^^,^^13^ At the same time, any mutation that reduces the hydrophobic interaction between the SWIRM and AOL domains will lead to a decrease in catalytic activity. The AOL domain of LSD1 (residues 271–417 and 523–833) is mainly composed of two functional lobes, one binds to FAD, and the other one can recognize and bind to the substrate. There is a huge active site cavity in the substrate-bind lobe, it is more open than other FAD-dependent amine oxidases.^10^^,^^11^^,^^16^^,^^17^ The cavity surface of the active site of LSD1 consists of 50 amino acid residues, which remain unchanged among different species. Inside the active cavity, four main pockets with different chemical properties can be used for the conjugate with side chains of substrate. The first pocket that contains the isoalloxazine ring of the FAD cofactor is the main catalytic center of LSD1. Isoalloxazine moiety is surrounded by residues including Val317, Gly330, Ala331, Met332, Val333, Phe538, Leu659, Asn660, Lys661, Trp695, Ser749, Ser760, and Tyr761, and most mutations of the amino acids that make up the active site resulted in reduced activity (Fig. 1B). These amino acids participate in the exact positioning of the FAD cofactor and substrate. The other pockets are involved in accommodating the amino acid histone tail of the substrate.^10^ The Tower domain (residues 417–523) of LSD1 is located in the middle of the two AOL domains, consisting of 100 amino acids, forming two antiparallel long helices that interacted via a short disordered loop; the two helices bind tightly to each other by hydrophobic interactions. The Tower domain is directly connected to the catalytic center of the AOL domain of LSD1, speculating that it may control the size of the chamber to regulate the activity of LSD1 to modulate its catalytic activity. There is a CoREST binding site on the Tower domain, which can mediate the interaction between LSD1 and CoREST, and serve as an adapter to recruit other proteins.^11^^,^^14^ In general, the structure of the Tower domain is necessary for the demethylase activity of LSD1.

Post-translational modification refers to the addition or removal of specific groups of amino acids to change the biochemical properties of proteins thereby regulating cellular life activities. More than 450 species of unique protein modifications have been identified, including phosphorylation, acetylation, methylation, SUMOylation, and ubiquitination. LSD1 acts as a demethylase, is involved in the post-translational modification process of H3K4 and H3K9, and regulates the expression of downstream genes. Meanwhile, studies have shown that there are a variety of post-translational modifications on LSD1 which ultimately affect the substrate recognition and catalytic activity.

## Phosphorylation of LSD1

**Figure 2:**
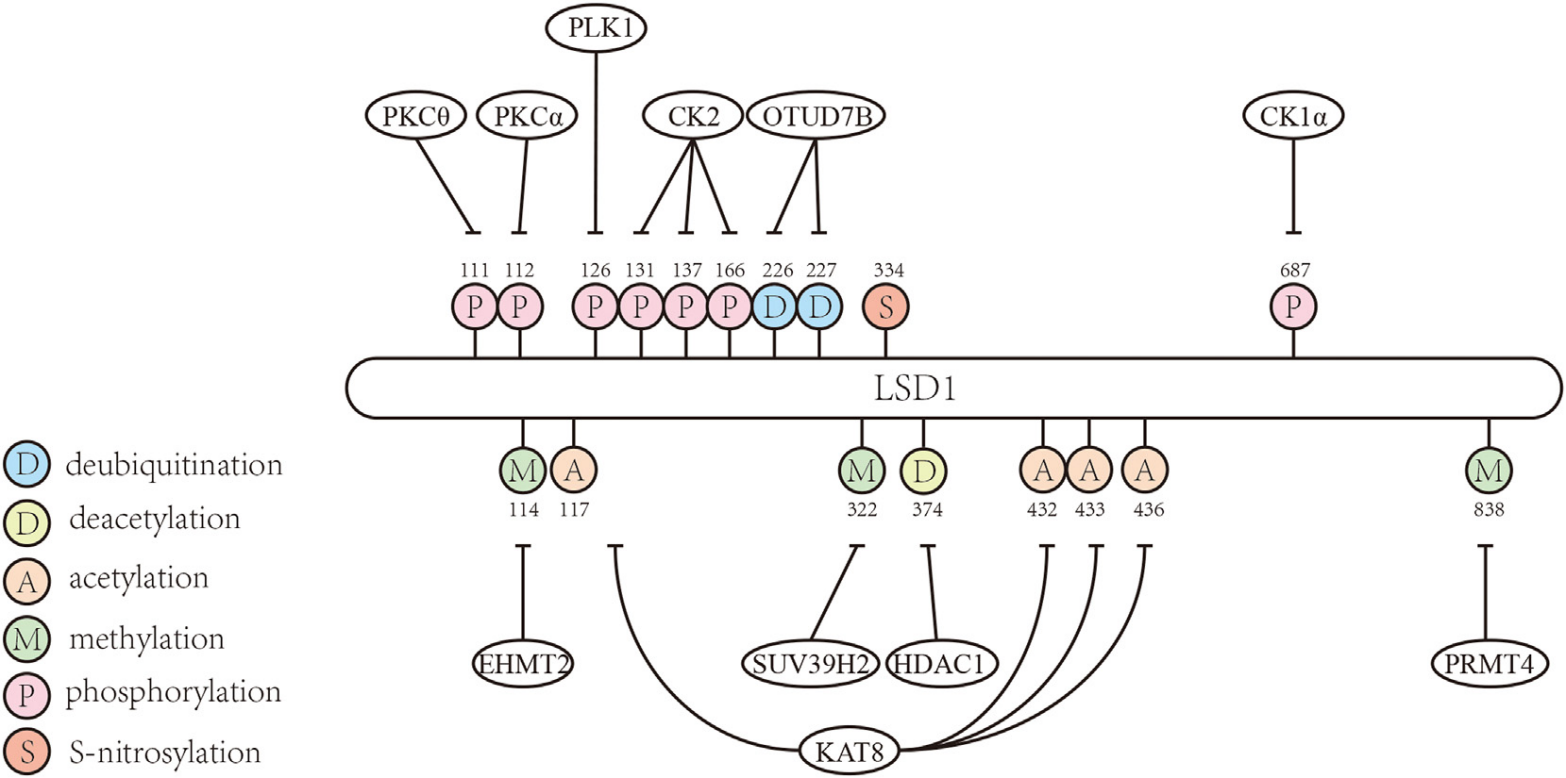
Post-translational modification sites of LSD1 and their associated modifying enzymes.

Phosphorylation refers to the process of transferring the γ phosphate group of ATP to the amino acid side chain of protein substrate catalyzed by protein kinases. PKC-θ (protein kinase c-θ) can directly phosphorylate LSD1 at amino acid 111, leading to the nuclear localization and the reduction of demethylase activity of LSD1 during epithelial–mesenchymal transition with changes of epithelial–mesenchymal transition markers vimentin and snail, which results in the gene repression induced by LSD1 demethylated H3K4 (Fig. 2).^18^ After modification at amino acid 111, PKC-α (protein kinase c-α) transfers to the nucleus to phosphorylate LSD1 at amino acid 112, phosphorylated LSD1 can recruit and bind to p65 thereby demethylating p65 (methylation at amino acid 314/315) to maintain p65 protein stability, followed by recruitment of C/EBP δ (CCAAT/enhancer-binding protein δ) to facilitate the continuation of the inflammatory response, and regulates oxidative stress. Meanwhile, phosphorylation of amino acid 112 controls the recruitment of LSD1 and p65 to promoters of target genes.^19^, ^20^, ^21^ Another study has shown that phosphorylated LSD1 can bind to the E-cadherin promoter region and regulate demethylation of methylated H3K4 to promote epithelial–mesenchymal transition; phosphorylation of LSD1 at ser112 is associated with the function of LSD1 in promoting breast cancer metastasis *in vivo*.^22^ In several experiments in mice, loss of phosphorylation of LSD1 at ser112 results in impairment of social memory, short-term memory, and hippocampus-dependent spatial memory. This illustrates phosphorylation of LSD1 is also required for contextual fear memory formation, presynaptic plasticity, and memory-related gene regulation.^23^ Furthermore, phosphorylation of LSD1 at serine 112 forms a complex with the CLOCK-BMAL1 (brain and muscle ARNT-like) heterodimers involved in E-box (CLOCK-BMAL1 E2-binding site)-mediated transcriptional activation. Defects in phosphorylation also lead to defective circadian rhythmicity, circadian rhythmicity phase resetting of the circadian clock, and impaired behavioral adaptation to photic stimuli in mice.^24^ LSD1 can locate centrosomes and spindle poles and is required for centrosome duplication and chromosome mis-segregation through regulating transcription of BUBR1 (BUB1 mitotic checkpoint serine) and MAD2 (mitotic arrest deficient 2). LSD1 is hyperphosphorylation during mitosis.^25^ In another study, it has been shown that PLK1 (POLO-like kinase 1) mediates the phosphorylation of LSD1 at amino acid 126, thereby promoting the release of LSD1 from chromatin during mitosis.^26^ STIP1 (stress-induced phosphorylated protein 1), a chaperone of Hsp90 (Heat shock protein 90), forms a STIP1-HSP90 complex that serves as a scaffold for GSK3β (glycogen synthase kinase-3 beta)-mediated phosphorylation of LSD1 (double-mutant at amino acid 707/711 of LSD1 hinder phosphorylation by GSK3β). It enhances the stability and subcellular localization of LSD1 and can promote cell proliferation.^27^ CK2 (casein kinase 2) is a ubiquitously expressed kinase in cells and is involved in the regulation of a variety of cellular activities with more than 500 substrates. CK2 is found to co-localize with LSD1 to the mitotic spindle. CK2-mediated phosphorylation of LSD1 amino acids at serine 131/137 not only promotes the interaction between LSD1 and RNF168 (RING finger 168) but also promotes RNF168-dependent 53BP1 (p53-binding protein 1) ubiquitination and enrichment of 53BP1 to response DNA damage. In conclusion, phosphorylation of LSD1 can promote cell proliferation and survival in genotoxic stress.^8^^,^^28^ Another kinase CK1α (casein kinase 1α) phosphorylates LSD1 at ser687, thereby promoting phosphorylation at ser683 of LSD1 by GSK3β to antagonize LSD1 ubiquitination, which can eventually maintain the development of glioma stem cells.^29^

Interestingly, there is a neurospecific isoform of LSD1 with two undescribed exons in the human hippocampus. The neurospecific isoform is longer (72 bp) and has more amino acids than LSD1. Neurospecific isoform still retains substrate-specific and demethylation activity.^30^ Further studies have shown that there is a phosphorylation site on the undescribed exon of neurospecific isoform. This phosphorylation induces HDAC and CoREST complex disassembly and modulates transcriptional repression in neurons.^31^

## Acetylation and deacetylation of LSD1

Acetylation refers to the process by which an acetyl group is transferred from the donor and added to a lysine or the N-terminus of a protein substrate catalyzed by acetyltransferase. Deacetylation is the opposite of acetylation. KAT8 (lysine acetyltransferase 8, also known as MOF) acetylates LSD1 at amino acids 432, 433, and 436 in epithelial cells, which reduces LSD1 binding with nucleosomes/chromatin. These residues direct contact with nucleosome DNA, and acetylation neutralizes their positive charge, eliminating electrostatic interactions with the negatively charged DNA phosphate backbone. This leads to the inhibition of the demethylation of H3K4, thereby resulting in suppression of tumor invasion and epithelial gene expression.^32^ Another study shows KAT8 also acetylates LSD1 at lysine K117, thus affecting the cytoplasmic localization and protein stability of LSD1 in liver cancer cells, thereby regulating the downstream FKBP8 (FKBP prolyl 0isomerase)-BCL2 (B-cell lymphoma-2) axis to promote tolerance of cellular to the drug and cell growth (Fig. 2).^33^ HDAC5 (histone deacetylase 5) and LSD1 coordinately overexpressed in breast cancer. HDAC5 exerts a deacetylation function through the nuclear localization sequence interacting with LSD1. The deacetylation stabilizes protein levels and promotes the catalyzed activity of LSD1 by preventing ubiquitination degradation. However, reducing HDAC5 alone does not increase LSD1 acetylation *in vivo*.^34^ Another deacetylase HDAC1 (histone deacetylase 1) deacetylates LSD1 at amino acid 374, affecting the binding of LSD1 to the H3 histone substrate and expression of target gene.^35^ Furthermore, deacetylase SIRT1 (sirtuin1), a key regulator of tumorigenicity in liver cancer stem cells, interacts and deacetylates LSD1 to adjust LSD1 stability. Eventually, the deacetylation can promote liver cancer stem cell self-renewal and tumorigenicity.^36^

## Ubiquitination and deubiquitination of LSD1

Ubiquitination refers to the process by which ubiquitin covalently binds to target proteins catalyzed by a series of enzymes. Ubiquitinated proteins can be recognized and degraded by the proteasome, reducing the expression level of the target protein within the cell. Deubiquitination is the process that removes ubiquitin from substrates catalyzed by deubiquitinating enzymes, eventually achieving the purpose of stabilizing protein levels. Jade-2 (jade family PHD finger 2), an E3 ubiquitin ligase for LSD1, promotes the polyubiquitination of LSD1 (interacting with the AOL/Tower domain of LSD1) through the C-terminal and the plant homology domain. In the experiment, it was found that the change in the expression level of LSD1 affects cortical progenitor differentiation and neuroectoderm induction. Thus, Jade-2 promotes neuroblastoma cell differentiation by targeting LSD1 degradation.^37^ RIP140 (receptor-interacting protein 140) interacts directly with the AOL domain of LSD1 through the RD1 domain (domain of RIP140, amino acid 78–333) and competes with Jade-2 to hinder the polyubiquitination of LSD1 and block subsequent degradation. At the same time, RIP140/LSD1 complex is recruited on the promoter region of Pax6 (paired box gene 6), thereby regulating neuronal differentiation.^38^ OTUD7B (OTU domain containing 7B) deubiquitinates K63-linked polyubiquitylated LSD1 (at amino acid 226/277) through the ubiquitin-associated domain and zinc finger domain, affecting the expression of downstream genes by regulating the stability of LSD1. The deubiquitination affects the transition of G1/S of the cell cycle. At the same time, the loss of OTUD7B promotes the binding of LSD1 to P62, a polyubiquitin chain binding protein, leading to LSD1 proteasomal degradation.^39^ USP28 (ubiquitin-specific protease 28) catalyzes deubiquitination of LSD1 through the N-terminal region of USP28 and the AOL domain of LSD1 to stabilize LSD1 protein, which enables direct regulation of the expression of differentiation genes, indirectly regulating the expression of the pluripotency activators.^40^ In glioma, a lack of methionine inhibits the growth of cancer cells and enhances the binding of LSD1 to E3 ubiquitin ligase CBL (casitas B-lineage lymphoma), and increased ubiquitination of LSD1 enhances expression of CXCL8 (CXC motif chemokine ligand 8) and methylated histone H3, which in turn affects the metabolism of the glycerophospholipid.^41^ USP7 (ubiquitin-specific protease 7) is able to interact with LSD1, exercising deubiquitination functions and enhancing the inhibitory effect of LSD1 on p53 (tumor protein p53).^42^, ^43^, ^44^ Phosphorylation of LSD1 catalyzed by GSK3β promotes the binding of LSD1 to USP22 (ubiquitin specific protease 22), and deubiquitination of LSD1 promotes tumor formation.^29^^,^^45^ USP38 (ubiquitin-specific protease 38) interacts with LSD1 at amino acid 454 to stabilize protein level. USP38 promotes the ability of cellular drug tolerance and colony formation in colon cancer cells by deubiquitinating LSD1.^46^

## Methylation of LSD1

Methylation is an important epigenetic modification modality that alters the structure and function of proteins by adding methyl groups to amino acid residues of substrate. In a study of breast cancer, PRMT4 (protein arginine methyltransferase 4) plays an important role in the process of USP7 deubiquitinating LSD1 by asymmetric demethylation of LSD1 at amino acid 838 (Fig. 2). PRMT4-mediated methylation promotes the stabilization of LSD1 to enhance cell migration and invasion, and the methylation levels are associated with the grade of breast carcinoma.^42^ SUV39H2 (suppressor of variegation 3–9 homolog 2) trimethylated LSD1 at amino acid 322, thereby LSD1 polyubiquitination was inhibited while also enhancing binding to CoREST.^47^ EHMT2 (euchromatic histone-lysine N-methyltransferase 2) can methylate LSD1 at amino acid 114. The methylation regulates the interaction between LSD1 and CHD1 (chromodomain-helicase-DNA-binding protein 1). EHMT2-LSD1-CHD1 axis controls the chromatin binding of AR (androgen receptor), and this pathway affects the expression of androgen-dependent genes and controls androgen-dependent chromosomal rearrangement.^9^

## SUMOylation of LSD1

SUMO (small ubiquitin-like modifier) is a newly discovered ubiquitin-like molecule. SUMOylation modifications are similar to ubiquitination. The C-terminus of SUMO is covalently bound to the lysine of the target protein and this process is driven by a series of enzymes, but the SUMOylation does not mediate the degradation of the target protein compared with ubiquitination. There is a direct, non-covalent interaction between CoREST and SUMO-2 (small ubiquitin-like modifier 2), and then CoREST bridges LSD1 binding to SUMO-2, with HDAC and LSD1 being recruited in a SUMO-dependent manner. In conclusion, SUMO-2 adjusts the CoREST/LSD1 complex on the downstream gene promoter region and results in transcriptional repression.^48^^,^^49^ Another study suggested that LSD1 was present in coimmunoprecipitation complex with SUMOylation of SATB2 (special AT-rich sequence-binding protein 2), and it also illustrates that SUMO-2 plays an important role in the exertion of demethylase activity of LSD1.^50^

## S-nitrosylation of LSD1

S-nitrosylation is a redox-dependent post-translational modification mediated by NO (nitric oxide), this modification is a non-enzymatically reversible process. Almost all proteins correlated with biological processes are subjected to S-nitrosylation. Aberrant expression of S-nitrosylation proteins is associated with many diseases, such as neurodegenerative diseases, and diabetes, and correlated with the initiation and maintenance of cancer. In a study of zebrafish, it was shown that the S-nitrosylation of LSD1 at amino acid 334 reduced its demethylase activity, resulting in the accumulation of methylated H3K4 in cells (Fig. 2). Thereby the expression of proangiogenic genes is promoted. Interestingly, the sequence 60 amino acids on both sides of amino acid 334 of LSD1 protein of zebrafish with the human ortholog retains 100% identity.^51^

In addition to the above mentioned, there are some post-translational modifications on different amino acids of LSD1 found by mass spectrometry and other methods in the project about normal cells and cancerous cells such as melanoma cells and breast cancer cells. These modifications have not been reported in the literature to find their associated factors, but still indicate that these positions possibly participate in cell life activities, and the different modifications of amino acid may be related to the progress of tumors (Table 1).

**Table 1:** The main post-translational modifications of LSD1.

Type of post-translational modification	Modification sites of LSD1
Phosphorylation	27,^52^ 59,^52^ 69,^52^, ^53^, ^54^, ^55^, ^56^, ^57^ 80,^55^ 88,^52^^,^^53^ 95,^55^ 97,^52^ 104,^52^^,^^54^^,^^55^^,^^58^ 135,^58^ 136,^56^^,^^58^^,^^59^ 172,^56^^,^^59^ 181,^59^ 466,^52^ 611,^56^ 841,^52^^,^^56^^,^^60^ 849,^52^^,^^55^, ^56^, ^57^^,^^59^, ^60^, ^61^, ^62^ 851,^52^^,^^56^^,^^59^, ^60^, ^61^ 852^52^
Ubiquitination and deubiquitination	268,^63^^,^^64^ 271,^64^ 280,^63^ 288,^65^ 322,^63^ 355,^63^^,^^64^ 359,^63^^,^^64^ 372,^63^ 374,^63^ 424,^63^^,^^64^ 436,^63^^,^^64^ 442,^63^^,^^64^ 447,^63^^,^^64^ 456,^63^^,^^64^ 463,^64^ 469,^63^^,^^64^ 471,^65^ 492,^63^ 503,^63^ 507,^63^^,^^64^ 617,^63^ 631,^63^ 647,^63^^,^^64^ 661,^63^ 732,^63^ 744,^63^ 768^65^
Methylation	187^66^
SUMOylation	117,^67^ 424,^67^ 442,^67^ 476,^68^ 609^68^

## LSD1 related tumors

**Figure 3:**
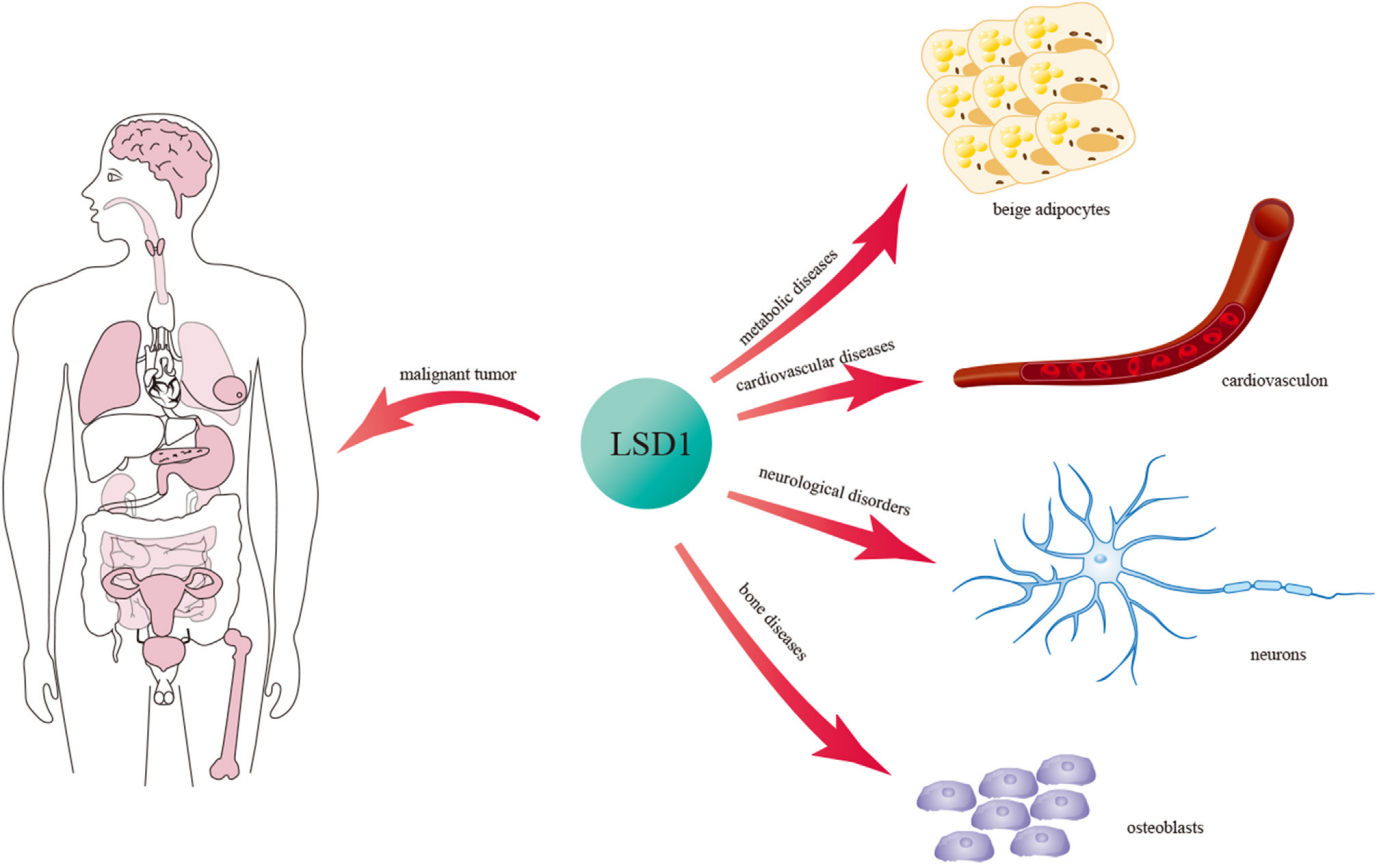
Diseases associated with LSD1.

As a histone demethylase, LSD1 is found abnormally expressed in a variety of cancer cells and is involved in tumorigenesis and metastasis (Fig. 3). In esophageal squamous cell carcinoma, LSD1 regulates the expression of downstream genes by demethylating the dimethylated H3K4 and then promotes the migration and invasion of esophageal squamous cell carcinoma.^69^ LSD1 recruited by Lnc00673l binds to the promoter regions of LATS2 (large tumor suppressor kinase 2) and KLF2 (Krüppel-like factor 2) and decreases methylation levels, thereby inducing growth arrest and decreased invasion of gastric cancer cells.^70^ SETD7 (SET domain containing 7) promotes ubiquitination degradation of RIOK1 (right open reading frame kinase 1) by methylation, while LSD1 stabilizes RIOK1 through demethylation. The high protein levels of RIOK1 stabilized by LSD1 promote the proliferation, invasion, and metastasis of colorectal cancer and gastric cancer cells, and correlate with poor overall survival.^71^ At the same time, LSD1 binds to the promoter region of TSPAN8 (tetraspanin 8) to demethylate the dimethylated H3K9 and increase expression of TSPAN8. Eventually, dysregulation of TSPAN8 affects the metastasis of colorectal cancer cells.^72^ LSD1 up-regulates LEF1 (lymphoid enhancer binding factor 1) through β-catenin to promote the epithelial–mesenchymal transition of bladder cancer and tumor progression.^73^ In kidney cancer, LSD1 interacts with AR and regulates downstream target genes of AR through demethylation of H3K9, and inhibition of LSD1 increases the sensitivity of kidney cancer cells to drugs.^74^ In cervical cancer, LSD1 is negatively correlated with GPER (G-protein coupled estrogen receptor), and high expression of LSD1 is related to the low lifetime of cervical cancer patients^75^; HIF1α (hypoxia-inducible factor-1α) is involved in the sensing and adaptation of cellular oxygen level, while in pancreatic cancer, LSD1 is positively correlated with HIF1α and prevents the acetylation-dependent degradation of HIF1α by recruiting HDAC2 (histone deacetylase 2) to maintain the stability of HIF1α.^76^ Hydroxylation and methylation are prerequisites for acetylation of HIF1α. Acetylation modification promotes ubiquitination degradation of HIF1α by enhancing the interaction between HIF1α and VHL(Von Hippel-Lindau). On the one hand, LSD1 can stabilize HIF1α by inhibiting SET9-mediated HIF1α methylation and PHD2 (prolyl hydroxylase domain protein 2)-mediated HIF1α hydroxylation. On the other hand, LSD1 enhances transcription and protein stability of MTA1 (metastasis associated 1) by demethylating H3K9 to enhance the activity of HDAC2, thereby increasing the deacetylation level of HIF1α. Ultimately, LSD1 can promote tumor angiogenesis by enhancing HIF1α/CBP/MTA1-dependent VEGF (vascular endothelial growth factor) transcription.^77^ At the same time, LSD1 is significantly related to the prognosis and clinicopathological condition of pancreatic cancer. After the knockdown of LSD1, it was found that the impaired expression of CCNA2 (cyclin A2) and the growth of pancreatic cancer is partially inhibited.^78^ Another study in thyroid cancer cells showed that LSD1 directly regulates APC2 (adenomatous polyposis coli 2) and indirectly promotes the transcription of DKK1 (dikkopf-related protein 1) through HIF1α. APC2 and DKK1 down-regulate β-catenin by enhancing ubiquitination levels to control the Wnt/β-catenin pathway, thereby promoting the progression of thyroid cancer and decreasing the sensitivity to chemotherapy.^79^ Pharmacological inhibition of LSD1 impairs the function of mitochondrial respiration and activates senescence in glioblastoma cells mediated by protein degradation of HIF1α.^80^ In neuroblastoma, Jade-2 acts as an E3 ubiquitin ligase to regulate the degradation of LSD1 and inhibit the differentiation of tumor cells.^37^ There is a high expression of LSD1 in osteosarcoma; histone methyltransferase SUV39H2 stabilizes LSD1, and then LSD1 regulates the methylation of CDH1 (cadherin 1, encoding E-cadherin) to repress the expression; transcription inhibition of CDH1 leads to the migration of osteosarcoma cells, thereby monitoring the progression of osteosarcoma.^81^ Similarly, there is a high expression of LSD1 in mantle cell lymphoma. LSD1 is positively correlated with Ki67. On the other hand, the inhibition of LSD1 impairs the proliferation and facilitates apoptosis of mantle cell lymphoma cells.^82^ LSD1 is overexpressed in non-small cell lung cancer and promotes proliferation and invasiveness of cancer cells. Studies have shown that LSD1 binds to the promoter region of TIMP3 (tissue inhibitors of metalloproteinases 3) to demethylate histone and block transcription of TIMP3, and results in the phosphorylation of JNK (Jun N-terminal kinase) and the expression of MMP2 (matrix metalloproteinases 2). The high expression of LSD1 and low expression of TIMP3 are also associated with poor prognosis of patients.^83^^,^^84^ At the same time, high expression of TdIF1 (TdT-interacting factor 1) in non-small cell lung cancer correlates with poor prognosis. Further studies confirm that TdIF1 regulates histone methylation levels by recruiting LSD1 on the promoter region of E-cadherin to affect cell migration and invasion.^85^ Similarly, the poor cardiovascular prognosis of patients with hypopharyngeal squamous cell carcinoma was also confirmed to be associated with high expression of LSD1.^86^ In prostate cancer, LSD1 binds to the promoter region of VEGF-A (vascular endothelial growth factor A) to regulate the protein level via AR, and VEGF-A partially promotes cell invasion and metastases by inducing epithelial–mesenchymal transition.^87^

## Metabolic diseases

LSD1 has also been found to play an important role in other diseases. In the metabolism of the human body, butyrate regulates adipose thermogenesis through LSD1 (Fig. 3).^88^ Mature adipocytes are classified into white adipocytes, brown adipocytes, and beige adipocytes based on function and morphology. White adipocytes mainly store energy in the form of fat. Brown and beige adipocytes respond to cold stimuli and produce heat. The adipocytes in epicardial adipose tissue are crucial to heart function, and the beige adipocytes in epicardial adipose tissue have the competence of adipose thermogenesis. LSD1 is involved in the differentiation from preadipocytes to beige adipocytes.^89^ In another experiment with mice of different ages, it was found that LSD1 was involved in the maintenance of growth and development of beige adipocytes by regulating Ppara (peroxisome proliferator-activated receptor α) and antagonizing the transition of beige adipocytes to white adipocytes.^90^ LSD1 inhibition can promote the differentiation of human embryonic stem cells to insulin-producing cells through ERK (extracellular regulated protein kinase) signaling, and improve the sensitivity of adipocytes to insulin, which may be a new strategy to improve obesity and treat diabetes.^91^^,^^92^ During the myogenesis, LSD1 preferentially binds to chromatin regions of oxidative-fiber-associated genes. The inhibition of LSD1 activates the oxidative metabolism of myotubes by enhancing the location of H3K4 at the target region.^93^ In trophoblast stem cells, the transcriptional regulation of LSD1 is found to be associated with metabolic processes and mortality/aging based on enrichment analyses. LSD1 plays a crucial role in cellular senescence by binding to the promoter region of Sirt4 (sirtuin 4), thereby governing glutamine anaplerosis, which is essential for maintaining redox balance and functional mitochondria.^94^

## Neurological disorders

LSD1 is essential to the survival of hippocampal and cortex neurons. The loss of LSD1 results in severe motor deficit, and impaired learning and memory in mice.^95^ Tauopathies is a type of neurodegenerative disease associated with pathological Tau (Fig. 3). Pathological Tau blocks LSD1 from nuclear export to cytoplasm to induce neurodegenerative diseases.^96^ LSD1 regulates the transcription of Egr1 (early growth response 1) by mediating demethylation of the dimethylated H3K4, while Egr1 plays a critical role in regulating sociability. Inhibition of LSD1 leads to rescue social deficits and repetitive behaviors of autism in mice models.^97^^,^^98^ Another study shows that the isoforms of LSD1 generated by RNA alternative splicing bind to SVIL (supervillin) to form a complex that regulates the demethylation levels of dimethylated H3K9 to affect the differentiation of human neurons.^99^ In animal models of amyotrophic lateral sclerosis, high expression of LSD1 was found in motor neuronal cells and functioned as a transcriptional repressor.^100^

## Cardiovascular diseases

During several vascular diseases, neointima formation is a major contributor to stenosis. LSD1 inhibits the expression of p21 (also known as cyclin-dependent kinase inhibitor 1) by regulating methylated H3K4 and promotes the proliferation of vascular smooth muscle cells.^101^ There is a high expression of LSD1 in atherosclerosis.^102^ Lnc000048 regulates the expression of MAP2K2 (mitogen-activated protein kinase 2) by inhibiting the demethylation activity of LSD1 to promote the phosphorylation level of ERK and ultimately affects the atherosclerosis process in mice.^103^ Another study showed that Hcy (homocysteine) increases the expression level of SNF5 (SWI/SNF-related matrix-associated actin-dependent regulator of chromatin subfamily B member 1) in macrophages of atherosclerotic plaque. SNF5 co-localizes with LSD1 in the nucleus and promotes expression of LSD1, and then promotes the mono-methylated H3K4 to regulate secretion of IL-1β (interleukin 1β). Finally, it aggravates the inflammatory response of macrophages and accelerates atherosclerosis.^104^ During the endothelial-to-hematopoietic transition, the complex of LSD1 and CoREST recruited by GFI1 and GFI1B (growth factor independence 1 and 1B) controls the generation of hematopoietic stem cells and blood progenitors.^105^

## Bone diseases

Osteoblasts are the main functional cells of bone formation. Osteoclasts correspond to osteoblasts in function, which are the main functional cells of bone resorption and play an important role in the growth and development of bone. Abnormal osteoclast function may lead to osteoporosis, bone metastases from cancer, osteosclerosis, *etc*. LSD1 mediated by RANKL (receptor activator of nuclear factor kappa B ligand) regulates bone resorption and F-actin formation in osteoclast (Fig. 3).^106^^,^^107^ Another study shows that LSD1 inhibits the osteoblast differentiation of human mesenchymal stem cells *in vitro*. LSD1 binds to promoter regions of Wnt7B (Wnt family member 7B) and BMP2 (bone morphogenetic protein 2) to directly regulate transcription. Increased Wnt7B and BMP2 with a deficiency of LSD1 promote osteoblast activity, thereby enhancing bone mass.^108^ In the human adipose-derived stem cells, miR-317 impairs osteogenesis by increasing HES1 (hairy and enhancer of split 1) and decreasing NOTCH1 (notch receptor 1). In this process, the loss of LSD1 can promote the expression of NOTCH1 and the decline of HES1.^109^ In summary, pharmacological inhibition of LSD1 could enhance the treatment of osteoporosis. On the other hand, LSD1 is able to inhibit RA (retinoic acid) signaling by suppressing ALDH1A2 (aldehyde dehydrogenase 1 family member A2). Deficiency of LSD1 decreases expression of SOX9 (transcription factor sox 9) through up-regulation of RA signaling to affect endochondral bone formation, thereby impairing bone fracture healing.^110^

In this section, we provide a brief overview of the diseases associated with LSD1 activity. Combined with our previous summary of the post-translational modification of LSD1, it seems to be a feasible approach to inhibit the development of related diseases by regulating the post-translational modifications of LSD1 to affect its activity and function.

## Conclusions and future perspectives

Post-translational modifications play a crucial role in modulating protein properties and increasing the functional diversity of the proteome. In this comprehensive review, our focus was on elucidating the structure, post-translational modifications, and the physiological and pathological roles of LSD1 in various human diseases. LSD1, a FAD-dependent histone demethylase belonging to the monoamine oxidase family, has been extensively studied. It exerts its transcriptional regulatory effects by forming complexes with CoREST, NuRD, CtBP, and other proteins, leading to the inhibition or activation of the target gene through interactions with AR, ERα, and other factors.

As an enzyme involved in protein modification, LSD1 undergoes various post-translational modifications that regulate its activity and function. Recent studies have revealed that LSD1 is involved in regulating diverse cellular activities and is associated with a wide range of diseases. For example, it has been reported that LSD1 ubiquitinated by E3 ligase Jade-2 is down-regulated via the ubiquitin-proteasome system, ultimately promoting the differentiation of neuroblastoma cells.^37^ Another study shown that LSD1 phosphorylated by GSK3β antagonizes ubiquitination.^29^ Notably, LSD1 is highly expressed in most malignant tumors, correlating with poor prognosis. Therefore, understanding the post-translational modifications of LSD1 is of utmost importance.

As mentioned earlier, FAD binds to the AOL domain of LSD1 as a cofactor and participates in the demethylation process. Most of the LSD1 inhibitors with good activity have been reported to be designed based on tranylcypromine, which hinders the demethylation process of LSD1 by binding to FAD.^111^, ^112^, ^113^, ^114^, ^115^ The changes in function and activity resulting from post-translational modifications at different amino acids of LSD1 have been outlined above. Is it possible to develop drugs with fewer side effects and stronger specificity on specific sites of post-translational modifications of LSD1 based on the molecular phenotype of different diseases? On the other hand, the relevant site and factors of post-translational modifications of LSD1, as mentioned in this review, are not fully understood. This is a research direction worth looking forward to in the future.

By comprehensively exploring the structural features, post-translational modifications, and disease-related roles of LSD1, this review provides valuable insights into the potential therapeutic implications of targeting LSD1 and its modifications in various diseases.

## Funding

This study was supported by the Basic Research of Medical Science and Technique Foundation of Henan Province, China (No. SBGJ202301004 to X.B.C.), the Key Project of the High Education from the 10.13039/501100009101Education Department of Henan Province, China (No. 22ZX008 to Y.C.Z.), the Youth Supporting Program from Henan Province, China (No. 2021HYTP060 to Y.C.Z.), the Youth Supporting Program from 10.13039/501100004605Zhengzhou University (No. JC202044046 to Y.C.Z.), the Science and Technology Project of Henan Province, China (No. 232102311179 to Y.G.), the 10.13039/501100001809National Natural Science Foundation of China (No. U21A20416, 82020108030 to H.M.L, 82103997 to B.W.), and the 10.13039/501100002858China Postdoctoral Science Foundation (No. 2021M692950 to B.W., 2021M702942 to L.J.Z.).

## Author contributions

Y.R.L. and B.W. performed the literature search and drafted the manuscript. Y.C.Z, H.Q.K, A.H., L.J.Z., N.J.G., H.M.L., A.M., M.M., and Y.G. reviewed and edited the original draft manuscript. X.B.C. and Y.G. conceived and designed the study and provided financial support. All authors read and approved the final manuscript.

## Conflict of interests

The authors declared no conflict of interests.
